# *Muangnua arborea*, a new semislug (Gastropoda, Stylommatophora, Helicarionidae, Durgellininae) from Loei Province, northeastern Thailand

**DOI:** 10.3897/zookeys.894.38327

**Published:** 2019-12-03

**Authors:** Chanidaporn Tumpeesuwan, Sakboworn Tumpeesuwan

**Affiliations:** 1 Department of Biology, Faculty of Science, Mahasarakham University, Kantharawichai District, Maha Sarakham, 44150 Thailand Mahasarakham University Maha Sarakham Thailand; 2 Palaeontological Research and Education Centre, Mahasarakham University, Kantharawichai District, Maha Sarakham, 44150 Thailand Mahasarakham University Maha Sarakham Thailand

**Keywords:** Durgellini, Loei, Na Haeo, new species, semi-slug, slug, taxonomy, Thailand

## Abstract

*Muangnua arborea* Tumpeesuwan & Tumpeesuwan, **sp. nov.**, is described, based on specimens deposited in the land snail collection of Mahasarakham University, Thailand. This species is the second described in the genus *Muangnua*, for which colour pictures of the living semislug in natural habitats, scanning electron microscope photos of the radula, and anatomy of the mature specimens were studied and presented for the first time for this genus. Keys to genera of Southeast Asian slug-like semislugs and species of *Muangnua* are provided.

## Introduction

*Muangnua* Solem, 1966 is a genus of helicarionid semislug with its shell reduced to a partially calcified cap having only a single remnant of coiling and that is completely covered by fused shell laps. The mantle lobes form a large cephalic shield reaching nearly to its eyes. Based on the external morphology, *Muangnua* looks similar to the genus *Parmarion*Fischer 1856. *Muangnua limax* Solem, 1966 is the sole species in the genus, which was collected by B. Degerbol on 8 November 1958 from Doi Suthep in Chiang Mai Province, northern Thailand, at 1100 m above mean sea level. Since its discovery, no additional information on the species has been published (Panha 1996; Vorajuk 2000; Hemmen and Hemmen 2001; Nabhitabhata 2009).

**Figure 1.**
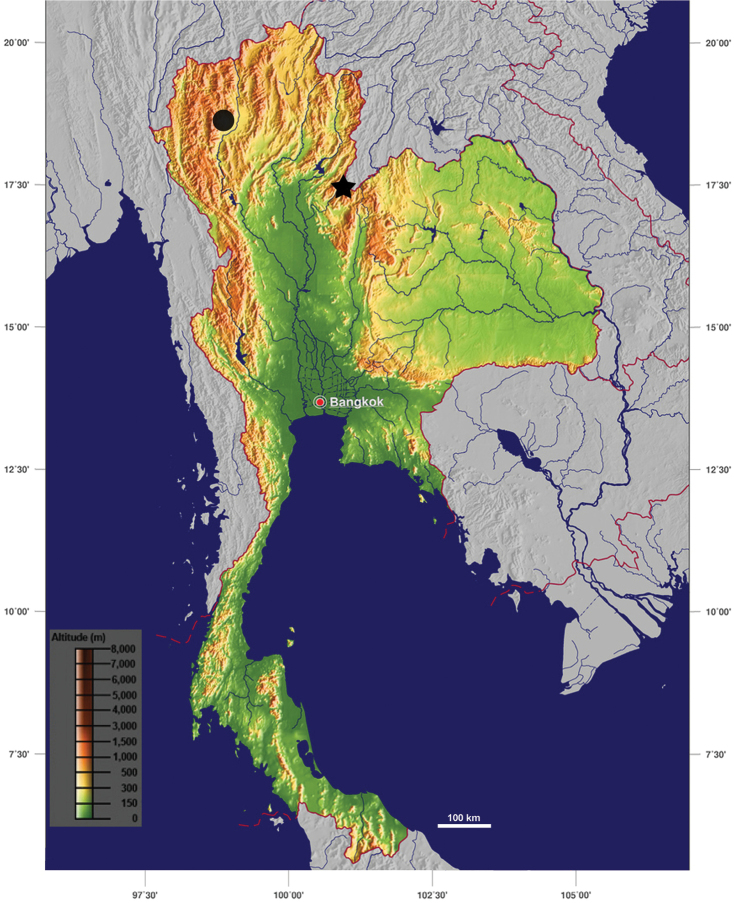
Map of type locality (circle is type locality of *Muangnua limax*; star is type locality of *Muangnua arborea* Tumpeesuwan & Tumpeesuwan, sp. nov.) (modified from GinkgoMaps−project http://www.ginkgomaps.com).

During the project ‘Landsnails of Na Haeo Area’, specimens and photographs of our previous expedition in Loei Province in 2011 were re-examined, and a second species of the genus *Muangnua* was discovered from the land snail assemblage of Phu Suan Sai sandstone mountain (Fig. 1), which is approximately 250 km southeast from Doi Suthep (the type locality of *M. limax*). This suggests that the genus has evolved at a high altitude and might be distributed over a wide area, and further new species in the genus from other high altitudes might be discovered in the future.

## Material and methods

**Figure 2.**
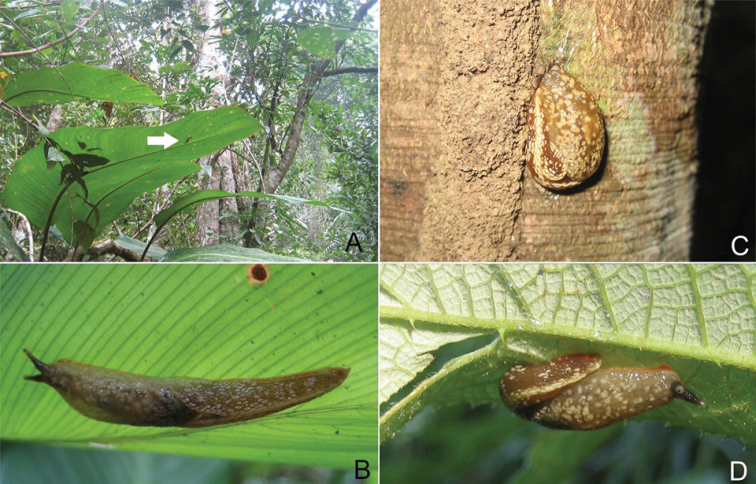
*Muangnua arborea* Tumpeesuwan & Tumpeesuwan, sp. nov. **A** in natural habitat, evergreen forest along small stream valley on sandstone mountain at type locality, in which new species was found on monocot leaf (indicated by white arrow) **B** close-up view of specimen (indicated by arrow in **A**) **C** new species in resting position on tree trunk **D** new species on dicot leaf.

**Figure 3.**
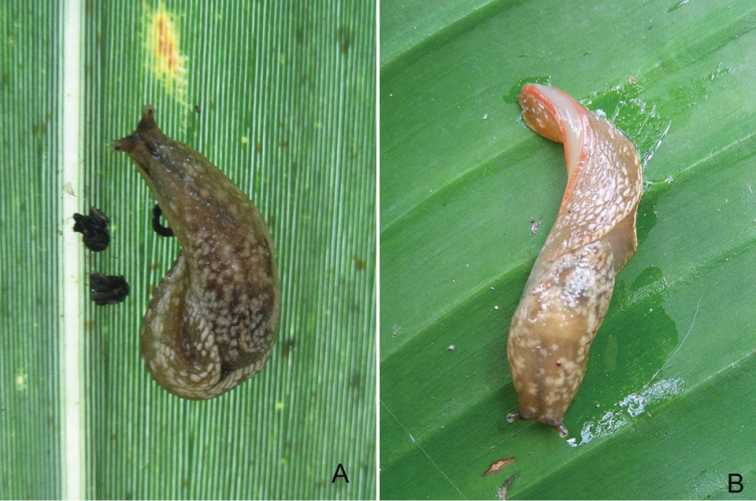
*Muangnua arborea* Tumpeesuwan & Tumpeesuwan, sp. nov. **A** in natural habitat, after waking from resting position on bamboo leaf **B** revealing protective behavior on banana leaf.

Living specimens were collected on 24–25 October 2011 from trunks, twigs, and leaves, etc. of monocotyledon and dicotyledon plants in an evergreen forest on sandstone hills at Phu Suan Sai, Na Haeo District, Loei Province, northeastern Thailand (Fig. 1). Snails were photographically documented in the natural sandstone habitat (Figs 2, 3), collected, drowned in water, and preserved in 70% ethanol to study their genital system and radula morphology. Adult shells were counted for whorl number and measured for shell height (SH) and shell width (SW), using digital vernier calipers. Adult snails were dissected to examine their genital system under a stereo microscope for description. Radula were extracted from the buccal mass and examined under a scanning electron microscope at the Centre for Scientific and Technological Equipment, Suranaree University of Technology, Thailand. The examined specimens were deposited in the land snail collection of the Natural History Museum, Mahasarakham University, Thailand (NHMSU).

## Results

### Order Stylommatophora A. Schmidt, 1855

#### Superfamily Helicarionoidea Bourguignat, 1877

**Family Helicarionidae Bourguignat, 1877**

**Subfamily Durgellinae Godwin-Austen, 1888**

**Tribe Durgellini Godwin-Austen, 1888**

##### Genus Muangnua

Taxon classificationAnimaliaStylommatophoraHelicarionidae

Solem, 1966

91717795-D060-5384-99A4-C3730EF5C0A4

###### Type species.

*Muangnua limax* Solem, 1966.

###### Diagnosis

(based on type material studied by Solem (1966)). Shell reduced, having only one remnant of coiling, which in living semislug is completely covered by fused shell laps. Mantle lobes form large cephalic shield reaching base of tentacles. Tail long and slender, with hooked caudal horn. Posterior visceral hump rounded, resting in V-shaped body groove. Jaw thin, without median projection or sculpture. Radula possesses tricuspid central, lateral, and marginal teeth. Genitalia, was studied from juvenile specimen, therefore, epiphallus cannot be differentiated. Free oviduct short, vagina rather long, dart apparatus, and other accessory penial organs absent; gametolytic sac (= spermatheca in Solem, 1966) long, finger-like, reaching two-thirds of way to albumen gland; atrium rather short.

###### Remarks.

The genus *Muangnua* was originally placed in the family Helicarionidae and tribe Durgelli by Solem (1966), after which Vaught (1989) moved it to the family Ariophantidae (Table 1). Panha (1996), Hemmen and Hemmen (2001), and Nabhitabhata (2009) followed Vaught’s classification. Schileyko (2002) also follows Vaught (1989), but he placed genus *Muangnua* into the subfamily Ariophantinae and tribe Ariophantini (Table 1). Recently, Bouchet and Rocroi (2005) and Bouchet et al. (2017) moved the tribe Durgellini into the subfamily Durgellinae (Helicarionidae). In this study, we followed Bouchet and Rocroi’s classification. Among the three genera of long elongate, small Southeast Asian semislugs (*Parmarion* P. Fischer, 1856; *Muangnua* Solem, 1966; and *Laocaia* Kuzminykh, 1999), their external morphology is very similar, but they possess many different characters. *Parmarion* frequently covers their ear-shaped shell with a mantle lobe, whereas *Muangnua* and *Laocaia* always cover their finger nail-shaped shell and triangular shaped shell, respectively, with their mantle lobe. The caudal horn overhangs in *Muangnua* and *Laocaia* but does not overhang in *Parmarion*. The postero-dorsal midline keel is present in *Parmarion* and *Muangnua* whereas it is present or absent in *Laocaia*.

**Table 1.** Classification of *Muangnua* Solem, 1966.

Authors	Taxa			
	Family	Subfamily	Tribe	Genus
Solem (1966)	Helicarionidae	Ariophantinae	Durgellini	* Muangnua *
Vaught (1989)	Ariophantidae	Macrochlamydinae	–	* Muangnua *
Panha (1996)	Ariophantidae	–	–	* Muangnua *
Hemmen and Hemmen (2001)	Ariophantidae	–	–	* Muangnua *
Schileyko (2002)	Ariophantidae	Ariophantinae	Ariophantini	* Muangnua *
Bouchet and Rocroi (2005)	Helicarionidae	Durgellinae	Durgellini	–
Nabhitabhata (2009)	Ariophantidae	Macrochlamydinae	–	* Muangnua *
Bouchet et al. (2017)	Helicarionidae	Durgellinae	Durgellini	–

##### Muangnua arborea

Taxon classificationAnimaliaStylommatophoraHelicarionidae

Tumpeesuwan & Tumpeesuwan sp. nov.

4D900D3E-D6EB-538F-A3E5-0FC35737CE06

http://zoobank.org/DE9258DC-692D-4AF1-891E-18C98D78EABC

Figs 2 , 3 , 4 , 5 , 6 , 7 , 8 , 9

###### Material examined.

**Figure 4.**
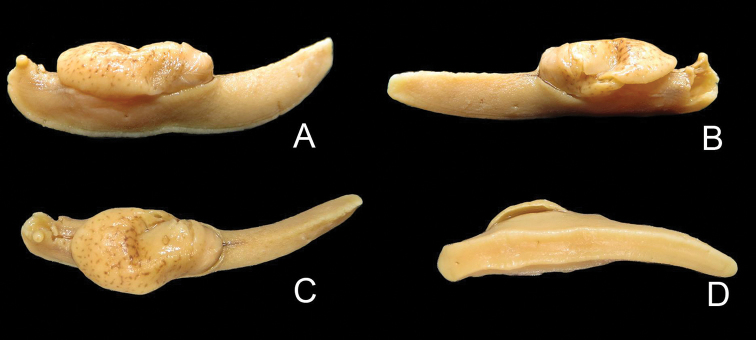
*Muangnua arborea* Tumpeesuwan & Tumpeesuwan, sp. nov. dissected holotype specimen NHMSU-00019 **A** lateral left **B** lateral right **C** dorsal **D** ventral. Photograph by: Benchawan Nahok.

***Holotype*.** (Fig. 4) Thailand: Loei Province, Phu Suan Sai sandstone mountain, in small valley of Suan Pa Na Po, type locality covered by evergreen forest with dense undergrowth of bamboo and banana, 17°27'55"N, 100°55'30"E; at 940–960 m above mean sea level, 24–25 October 2011; C. Tumpeesuwan, S. Tumpeesuwan, and member of MSU malacology laboratory leg.; NHMSU-00019. ***Paratypes*.** Seven adults and three juveniles, same data as for holotype: NHMSU-00020.

###### Etymology.

Specific epithet “arborea” derived from Latin word “arboreus” meaning “of trees” referring to the habitat of this new semislug species.

###### Differential diagnosis.

**Figure 5.**
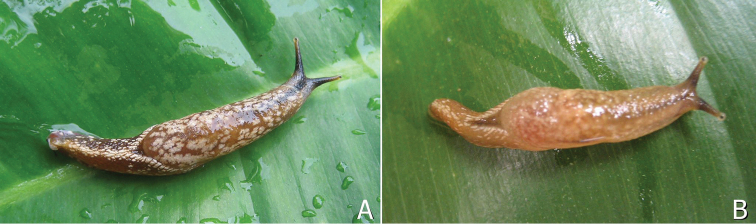
*Muangnua arborea* Tumpeesuwan & Tumpeesuwan, sp. nov. external morphology and coloration of body with visceral hump and V-shaped dorsal groove, **A** mature snail **B** immature snail.

**Figure 6.**
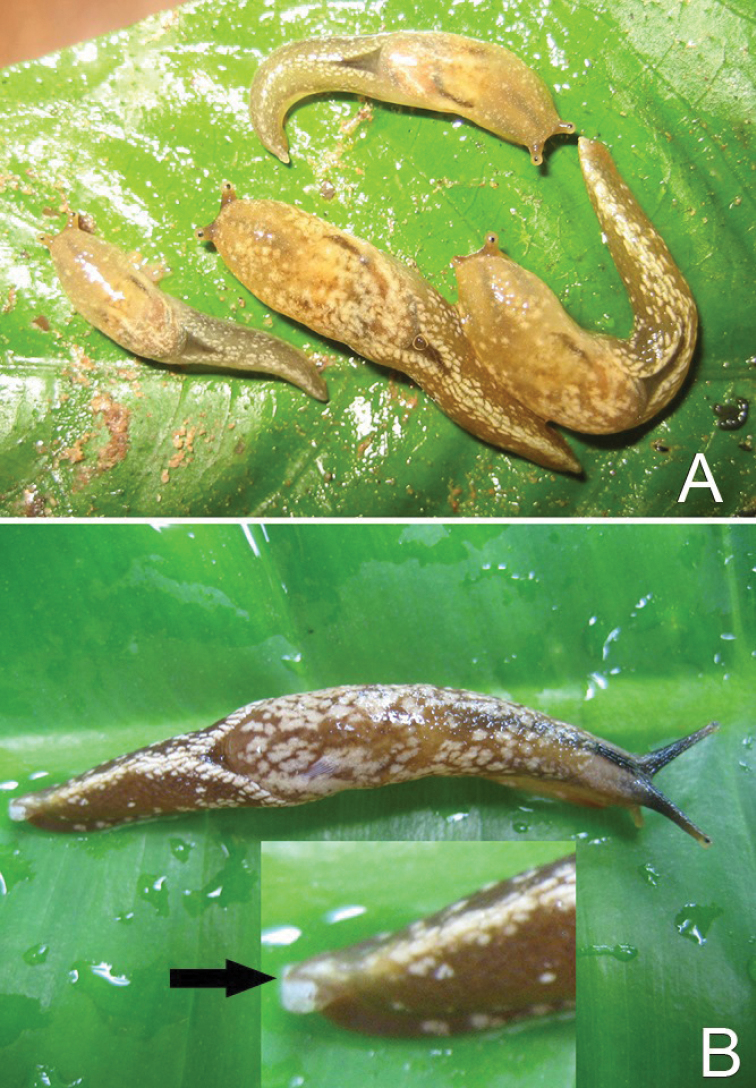
*Muangnua arborea* Tumpeesuwan & Tumpeesuwan, sp. nov. **A** colour pattern variation within population **B** close-up view of caudal horn (indicated by arrow).

(Table 2) *Muangnua arborea* Tumpeesuwan & Tumpeesuwan, sp. nov. differs from *Muangnua limax* Solem, 1966 by its body coloration. Head of *M. arborea* with three black or pale black strips, for which each lateral side of head possesses lighter stripes from base of lower tentacle back to base of cephalic shield, a darker mid-dorsal line from anterior extremity among tentacles back to one-third of body length under cephalic shield (Figs 3–6), whereas, mid-dorsal line black strips absent in *M. limax*. There are various sizes of white spots crowded on fringe and keel of living *M. arborea*, which causes remarkable white “Y” stripe on postero-dorsal side of foot (missing in preserved specimens), whereas, preserved specimen of *M. limax* possesses three black strips on postero-dorsal side of foot, a darker stripes on mid-dorsal keel, and two lighter stripes on both lateral sides.

**Table 2.** Comparison of morphological characters between *Muangnua limax* Solem, 1966 and *Muangnua arborea* Tumpeesuwan & Tumpeesuwan sp. nov.

Character	Taxa	
	*M. limax*	*M. arborea*
**Body**		
Mid-dorsal line	Absent	Present
Lateral stripe	Present	Absent
**Radula**		
Rows	120	> 123
Teeth/row	179	> 44
**Genitalia**		
Free oviduct	Present (short)	Absent
Vagina	Rather long	Very long
Gametolytic sac	Reaching two-thirds way to albumen gland	Reaching about half way to albumen gland

###### Description.

***Body***: Body is slender, elongated (Figs 2–6). Body length 37.1–45.3 mm and body width 10.2–10.4 mm when slightly retracted. Foot narrow, posterior part of foot laterally depressed and forms steep keel structure (Figs 2B–D, 3–6) tapering posteriorly. Tail long with hooked, caudal horn (Fig. 6B), tail length almost equal to half of length of posterior end of visceral hump to anterior end of head. Posterior lobe of visceral hump rounded, resting in “V” shaped body groove on top of foot (Figs 5, 6). Mantle lobes and shell laps fused and forming large cephalic shield, completely covering shell and visceral hump.

***Coloration***: Primary color of anterior body is light-ocher-brownish and gradually changes to brown or dark brown at posterior of body. Head possesses two pairs of tentacles (=ommatophores in Dedov et al. 2019). Lower tentacle is short and same color as head. Upper tentacle is black colored at base and gradually changes to light-ocher-brownish at top of tentacle, which contrasts with black eye spot on top of tentacle. Head with three black or pale black strips, for which each side of head possesses lighter stripes from base of lower tentacle back to base of cephalic shield and under its edge, a darker mid-dorsal line from anterior extremity among both tentacles back to one-third of body length under cephalic shield (Figs 3–6). Whole body has pattern of irregularly-dispersed various-sized white spots. Visceral hump with two short vague, pale brown lateral strips (Figs 3A, 5, 6A). Posterior lobe of visceral hump sitting in dark brown to pale black V-shaped depression on postero-dorsal side of foot. Fringe of V-shaped depression connects to steep keel on postero-dorsal side of foot. Various sizes of white spots crowded on fringe and keel, which causes remarkable white “Y” stripe on postero-dorsal side of foot; posterior extremity of this white Y-shaped stripe connects to white caudal horn.

**Figure 7.**
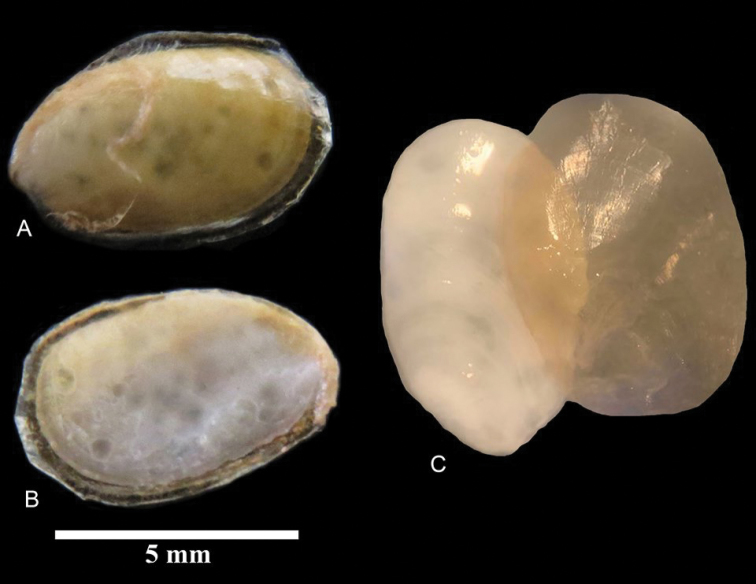
Shell of *Muangnua arborea* Tumpeesuwan & Tumpeesuwan, sp. nov. (paratype NHMSU-00020) **A** external view **B** internal view **C** periostracum removed from shell of preserved specimen.

***Shell*** (Fig. 7): Reduced to partially calcified cap having no coiling, shell length 7.1–7.4 mm, SW 5.1–5.5 mm., apex not prominent, shell shape similar to human finger nail, white calcified plate covered by transparent pale brown periostracum, normally sloughs off in preserved specimens (Fig. 7). In living semislug, its shell is always completely enclosed by fused shell laps.

**Figure 8.**
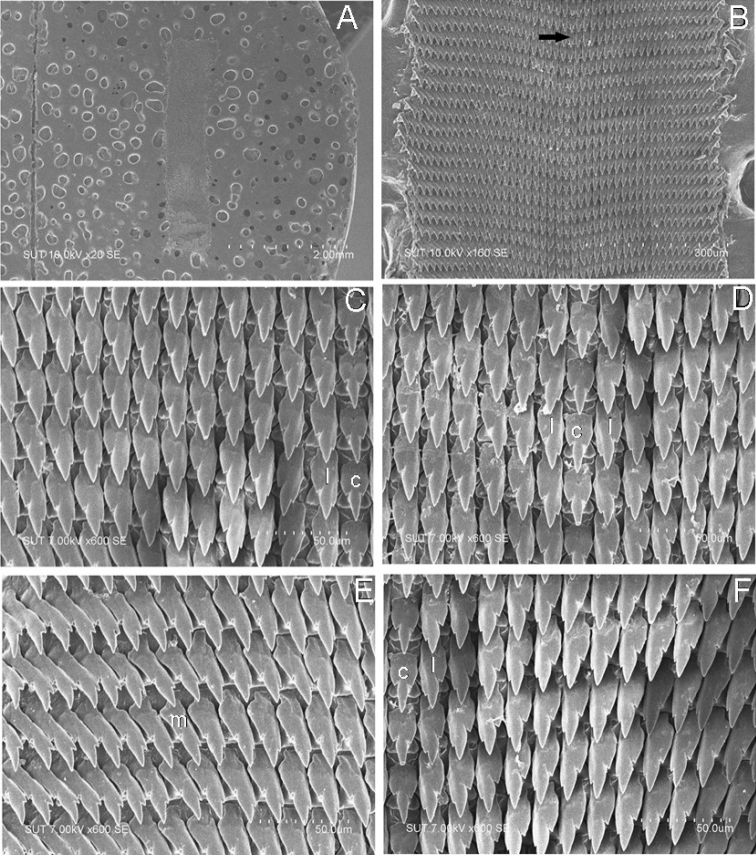
Radula morphology of *Muangnua arborea* Tumpeesuwan & Tumpeesuwan, sp. nov. (paratype NHMSU-00020) **A** radula plate (ribbon-like radula in middle of picture) **B** central tooth (arrow) and lateral teeth **C** close-up view of left side of radula **D** close-up view of middle part of radula **E** close-up view of left side of radula showing marginal teeth **F** close-up view of right side of radula.

***Radula*** (Fig. 8): According to old specimens preserved since 2011, edge of radula plates macerated and breaks down during radula extraction process. Although, we choose the best specimens from several individuals, nevertheless, most of the marginal teeth were lost or incomplete. Radula plate long ribbon-shape, comprised of at least 123 rows of teeth. Each row composed of more than 44 teeth. Central teeth isosceles triangle, tricuspid, very large broad base, mesocone long lancelate, ectocones prominent but short, not reaching nearly to edge of base plate. First lateral teeth with small entocone appearing on mesocone shaft, ectocone large and plump on outer side of cusps. Laterals have basal plate elongated, increasing as entoconal size increases and ectocone rapidly decreases in size. By 20^th^ tooth, teeth sub-equally bicuspid, as entocone becomes smaller than mesocone and ectocone reduced to a small side cusp.

**Figure 9.**
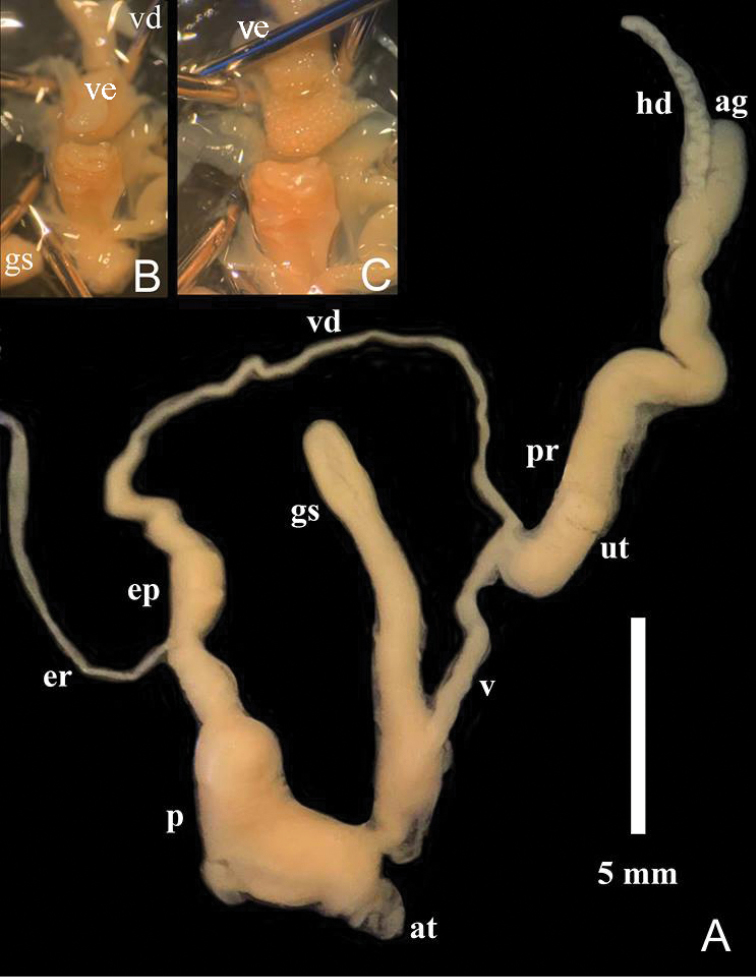
Reproductive anatomy of *Muangnua arborea* Tumpeesuwan & Tumpeesuwan, sp. nov. (paratype NHMSU-00020), **A** genitalia **B** inner part of penis **C** after verge inverted. Abbreviations: hd = hermaphroditic duct, ag = albumen gland, vd = vas deferens, ep = epiphallus, er = epiphallic retractor, p = penis, ve = verge, at = atrium, gs = gametolytic sac, ut = uterus, v = vagina, pr = prostate gland.

***Genitalia morphology*** (Fig. 9): Atrium rather short. Penis large and stout, peanut-shape, internally with short cylindrical muscular verge, which attaches to inner surface of penis at proximal end (connected to distal end of epiphallus); inner surface sculpture of penis can be divided into 2 types: upper portion around verge covered with numerous small tubercles, and lower portion covered with transverse folds (Fig. 9B, C); epiphallus length equal to penis, dumbbell-shape; vas deference inserts to epiphallus apically. Vagina very long and slender tube, prostate gland very small and encloses uterus, albumen gland small, hermaphroditic duct convoluted. Dart apparatus (amatorial organ) and other accessory penial organs absent. Gametolytic sac long, finger-like, reaching about half way to albumen gland.

###### Discussion.

According to the most recent information, this species is known only from the type locality. They were found only on plants (Fig. 2), which is normally on the leaves of monocot plants, such as bamboo, banana, etc. (Fig. 3). In the resting stage, this animal normally holds onto the leaves or trunks of plants by inverting their tail and attaching its end to the left side of their head, making their body U-shaped (Fig. 2C). This new species has a similar protective behavior to that described by Dedov et al. (2019) for *Laocaia simovi* and Wiktor (2002) for *Cryptaustenia saltatoria* and *Cryptaustenia obesa*. If they are touched or caught, they will quickly flip, wag, and twist their foot to escape from the predator (Fig. 3B). In addition, for some semislugs in the resting stage, we found them near their fecal matter (Fig. 3A), which might suggest this animal has homing behavior, and they will return to sleep in the same position every day.

According to Schilthuizen and Liew (2008) a semislug is defined as snails with a partially visible shell that, due to their rather small shell, cannot withdraw its body into its shell. *Muangnua* is a slug-like semislug possessing a reduced shell having only one remnant coil that is always covered by a mantle lobe. There are many species of semislug described and recorded from South and Southeast Asia (Blanford and Godwin-Austen 1908), including, *Girasia* Gray, 1855 from Himalaya and Assam (India), *Cryptogirasia* Cockerell, 1898 from Naga Hills (India), from Western Ghat (India) and Ceylon (Sri Lanka), *Austenia* Nevill, 1878 from the Himalaya, Assam (India) and Burma (Myanmar), *Parmarion* P. Fischer, 1856 from South China to Java (Indonesia), *Minyongia* Godwin-Austen, 1916 from Assam (India), and *Myotesta* Collinge, 1901 from North Vietnam. Of these genera, only *Austenia* and *Parmarion* are known in Thailand. *Austenia doisutepensis* Solem, 1966 has a short body and 1⅔ to 2 whorls; therefore, its mantle lobes and shell laps cannot cover all the shell surface and leave much of the shell exposed (snail-like semislugs). *Parmarion martensi* Simroth, 1893 has an elongated body, in which the small ear-shape reduced shell is frequently covered by mantle lobes and shell laps (slug-like semislugs). We provide below keys for identifying the genera of slug-like semislugs and species of *Muangnua* in Southeast Asia.

#### Key to genera of Southeast Asian slug-like semislugs

**Table:** 

1	Ear-shaped shell frequently covered by mantle lobe; Caudal horn not overhanging	*** Parmarion ***
–	Finger nail or triangular shape shell always covered by mantle lobe; Caudal horn overhanging	**2**
2	Finger nail-shaped shell; gametolytic sac long cylindrical tube	*** Muangnua ***
–	Triangular shaped shell with thin seam periostracum; gametolytic sac stalk short and stout or moderately long and slender	*** Laocaia ***

### Key to species of genus *Muangnua*

**Table:** 

1	Head without mid-dorsal line; tail with lateral stripe	***Muangnua limax***
–	Head with mid-dorsal line; tail without lateral stripe	***Muangnua arborea* sp. nov.**

## Supplementary Material

XML Treatment for Muangnua

XML Treatment for Muangnua arborea
